# Recent developments in classification criteria and diagnosis guidelines for idiopathic inflammatory myopathies

**DOI:** 10.1097/BOR.0000000000000549

**Published:** 2018-09-28

**Authors:** Alexander Oldroyd, Hector Chinoy

**Affiliations:** a NIHR Manchester Musculoskeletal Biomedical Research Centre, Manchester University Hospitals NHS Foundation Trust, Manchester Academic Health Science Centre; b Centre for Musculoskeletal Research, Manchester Academic Health Science Centre, The University of Manchester, Manchester; c Rheumatology Department, Salford Royal NHS Foundation Trust, Manchester Academic Health Science Centre, Salford, UK

**Keywords:** classification, dermatomyositis, diagnosis, myositis, polymyositis

## Abstract

**Purpose of review:**

The aim of this review was to summarize key developments in classification and diagnosis of the idiopathic inflammatory myopathies (IIMs).

**Recent findings:**

The recently published European League Against Rheumatism/American College of Rheumatology (EULAR/ACR) classification criteria for the IIMs provide a comprehensive, accurate and data-driven approach to identification of IIM cases appropriate for inclusion in research studies. Further, recent studies have advanced understanding of clinical manifestations of the IIMs and delineated the role of imaging, particularly magnetic resonance.

**Summary:**

The recent publication of the EULAR/ACR classification criteria will potentially greatly improve IIM research through more accurate case identification and standardization across studies.

Future inclusion of newly recognized clinical associations with the MSAs may further improve the criteria's accuracy and utility. Clear and comprehensive understanding of associations between clinical manifestations, prognosis and multisystem involvement can aid diagnostic assessment; recent advances include delineation of such associations and expansion of the role of imaging.

## INTRODUCTION

The idiopathic inflammatory myopathies (IIMs) are a group of autoimmune diseases characterised by chronic muscle inflammation (myositis), internal organ inflammation and significant morbidity and mortality [1]. The wide spectrum of clinical manifestations, variable disease course and distinct subtypes makes accurate classification and diagnosis of paramount importance to ensure valid research and timely instigation of treatment.

This article aims to summarize recently published research pertinent to advances in IIM classification and diagnosis. A Medline search for research articles published between January 2017 and May 2018 was carried out using the MeSH term ‘myositis’. Articles primarily focussing on myositis-specific autoantibodies were excluded, as they will be reviewed in detail in a separate article. 

**Box 1:**
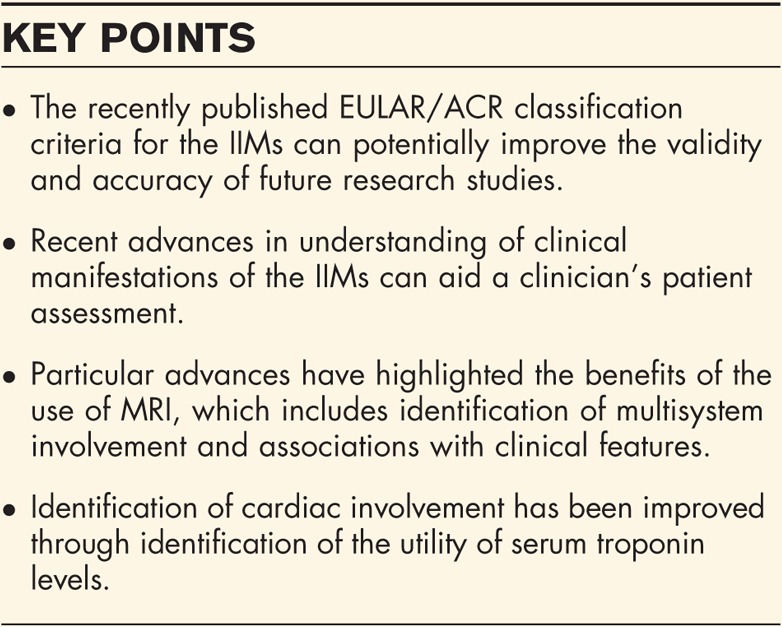
no caption available

## CLASSIFICATION

The IIMs have traditionally been classified and diagnosed according to the criteria by Bohan and Peter (Table 1) since publication in 1975 [2,3]. The Bohan and Peter criteria demonstrate a high degree of accuracy and usefulness in research and clinical settings. However, this usefulness is limited by a number of factors, including lack of specification of how to exclude other forms of myopathy and nonexplicit definition of inclusion criteria; further, recent advances in myositis research, such as the identification of myositis-specific autoantibodies (MSAs), are not included. Another major drawback of the Bohan and Peter criteria is the noninclusion of more recently described and defined IIM subtypes, including immune-mediated necrotising myopathy (IMNM) and antisynthetase syndrome (ASS).

**Table 1:** Bohan and Peter classification criteria for polymyositis and dermatomyositis

Criteria	Description
A	Proximal and symmetrical muscle weakness of the pelvic and scapular girdle, anterior flexors of the neck, progressing for weeks to months, with or without dysphagia or involvement of respiratory muscles
B	Elevation of the serum levels of skeletal muscle enzymes: creatine kinase, aspartate aminotransferase, lactate dehydrogenase and aldolase
C	Electromyography characteristic of myopathy (short and small motor units, fibrillation, positive pointy waves, insertional irritability and repetitive high-frequency firing)
D	Muscle biopsy showing necrosis, phagocytosis, regeneration, perifascicular atrophy, perivascular inflammatory exudate
E	Typical cutaneous changes:(1) Heliotrope rash with periorbital oedema and violaceous erythema(2) Gottron's sign: vasculitis in the elbow, metacarpophalangeal and proximal interphalangeal joints
Polymyositis	(1) Definite – all of A–D(2) Probable – any three of A–D(3) Possible – any two of A–D
Dermatomyositis	(1) Definite – E plus and three of A–D(2) Probable – E plus and two of A–D(3) Possible – E plus and one of A–D

Exclusion criteria: congenital muscular dystrophies, central or peripheral neurological disease, infectious myositis, metabolic/endocrine myopathies and myasthenia gravis. Adapted with permission [2].

These drawbacks have limited accurate identification of well defined populations suitable for IIM research studies. Therefore, in 2004, the International Myositis Classification Criteria Project (IMCCP) was established with the aim of developing new IIM classification criteria. The IMCCP working committee was formed of experts from adult and paediatric rheumatology, neurology, dermatology, epidemiology and biostatistics. In 2017, the newly developed European League Against Rheumatism/American College of Rheumatology (EULAR/ACR) classification criteria were published [4^▪▪^]. Initial methodology identified 93 candidate variables for inclusion in the classification criteria. Variable domains included pattern of weakness, dermatological manifestations, disease course, systemic manifestations, response to treatment, pattern of muscle biopsy abnormalities, presence of MSAs, electromyogram (EMG) and MRI features.

Using data from 976 IIM cases and 624 comparators, the combination of candidate variables that could most accurately distinguish between IIM and non-IIM participants was identified. The variables included in the final classification criteria are displayed in Table 2. Each variable was assigned a weighted score, according to the coefficient from a logistic regression model. The sum of the scores that a potential study participant fulfils corresponds to their probability of having an IIM. Without muscle biopsy data, a score between 5.5 (55% probability) and 7.5 (90% probability) corresponds to ‘probable IIM’ and a score equal to or greater than 7.5 corresponds to ‘definite IIM’. With biopsy data, a score between 6.7 (55% probability) and 8.7 (90% probability) corresponds to ‘probable IIM’ and a score equal to or greater than 8.7 is ‘definite IIM’.

**Table 2:** The European League Against Rheumatism/American College of Rheumatology classification criteria for adult and juvenile idiopathic inflammatory myopathies

			Score points	
Domain	Feature	Definition	With muscle biopsy data	Without muscle biopsy data
Age of onset	Age of onset of first symptom assumed to be related to the disease ≥18 and <40 years		1.5	1.3
	Age of onset of first symptom assumed to be related to the disease ≥40 years		2.2	2.1
Weakness pattern	Objective symmetric weakness, usually progressive, of the proximal upper extremities	Weakness of proximal upper extremities as defined by manual muscle testing or other objective strength testing, which is present on both sides and is usually progressive over time	0.7	0.7
	Objective symmetric weakness, usually progressive, of the proximal lower extremities	Weakness of proximal lower extremities as defined by manual muscle testing or other objective strength testing, which is present on both sides and is usually progressive over time	0.5	0.8
	Neck flexors are relatively weaker than neck extensors	Muscle grades for neck flexors are relatively lower than neck extensors as defined by manual muscle testing or other objective strength testing	1.6	1.9
	In the legs, proximal muscles are relatively weaker than distal muscles	Muscle grades for proximal muscles in the legs are relatively lower than distal muscles in the legs as defined by manual muscle testing or other objective strength testing	1.2	0.9
Skin manifestations	Heliotrope rash	Purple, lilac-coloured or erythematous patches over the eyelids or in a periorbital distribution, often associated with periorbital oedema	3.2	3.1
	Gottron's papules	Erythematous to violaceous papules over the extensor surfaces of joints, which are sometimes scaly. May occur over the finger joints, elbows, knees, malleoli and toes	2.7	2.1
	Gottron's sign	Erythematous to violaceous macules over the extensor surfaces of joints, which are not palpable	3.7	3.3
Other clinical manifestations	Dysphagia or oesophageal dysmotility	Difficulty in swallowing or objective evidence of abnormal motility of the oesophagus	0.6	0.7
Laboratory results	Anti-Jo-1 positivity	Autoantibody testing in serum performed with standardized and validated test, showing positive result	3.8	3.9
	Elevated serum levels of CK or LDH or AST or ALT	The most abnormal test values during the disease course (highest absolute level of enzyme) above the relevant upper limit of normal	1.4	1.3
Muscle biopsy features	Endomysial infiltration of mononuclear cells surrounding, but not invading, myofibers	Muscle biopsy reveals endomysial mononuclear cells abutting the sarcolemma of otherwise healthy, nonnecrotic muscle fibres, but there is no clear invasion of the muscle fibres	1.7	
	Perimysial and/or perivascular infiltration of mononuclear cells	Mononuclear cells are located in the perimysium and/or located around blood vessels (in either perimysial or endomysial vessels)	1.2	
	Perifascicular atrophy	Muscle biopsy reveals several rows of muscle fibres, which are smaller in the perifascicular region than fibres more centrally located	1.9	
	Rimmed vacuoles	Rimmed vacuoles are bluish by haematoxylin and eosin staining and reddish by modified Gomori trichrome stain	3.1	

ALT, alanine transaminase; AST, aspartate transaminase; CK, creatine kinase; LDH, lactate dehydrogenase. Adapted with permission [4^▪▪^].

**FIGURE 1:**
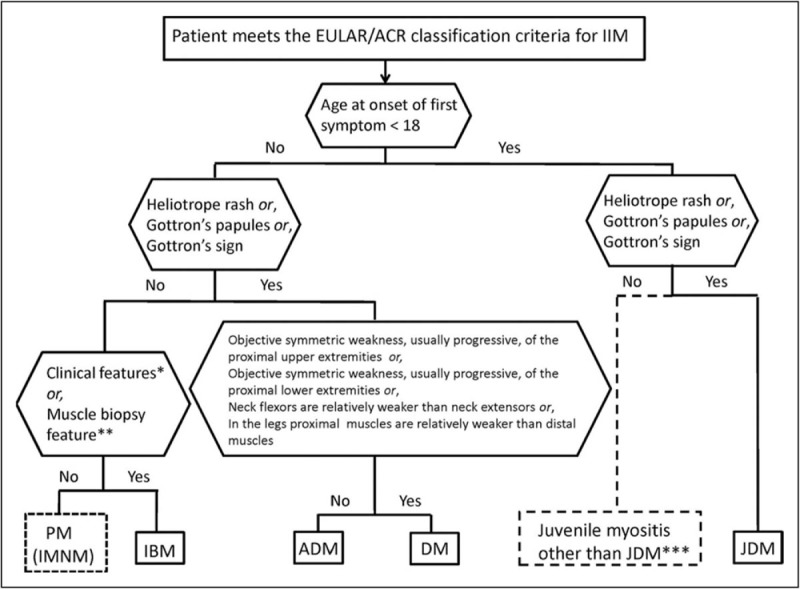
Classification tree for subtype of idiopathic inflammatory myopathies. ADM, amyopathic dermatomyositis; DM, dermatomyositis; EULAR/ACR, European League Against Rheumatism/American College of Rheumatology; IBM, inclusion body myositis; IMNM, immune-mediated necrotising myopathy; JDM, juvenile dermatomyositis; PM, polymyositis. For inclusion body myositis (IBM) classification, one of the following is required for classification: finger flexor weakness and response to treatment: not improved (^a^), or muscle biopsy: rimmed vacuoles (^b^). ^c^Juvenile myositis other than juvenile dermatomyositis was developed based on expert opinion. Adapted with permission [4^▪▪^].

The likely IIM subtype of each case can also be ascertained, according to the published classification tree (Fig. 1). Subtype identification can only be carried out on cases with an IIM diagnostic probability of greater than 55%. Further, only those with polymyositis, inclusion body myositis (IBM), dermatomyositis, amyopathic dermatomyositis (ADM) and juvenile dermatomyositis (JDM) can be identified, in part due to the small number of cases of IMNM, hypomyopathic dermatomyositis, ASS and overlap myositis cases in the development population.

The EULAR/ACR criteria demonstrated high sensitivity (93%) and specificity (88%) when muscle biopsy data were included. Sensitivity and specificity also remained high when muscle biopsy data were not included: 87 and 82%, respectively. The accuracy of the newly created classification criteria was compared against previously developed criteria, including those by Bohan and Peter [2,3], Tanimoto *et al*. [5], Targoff *et al*. [6], Dalakas and Hohlfeld [7] and the European Neuromuscular Centre [8]. The Targoff criteria demonstrated slightly higher accuracy with 93% sensitivity and 89% specificity. No other criteria showed both higher sensitivity and specificity. Also, the new criteria demonstrated correct IIM subtype classification in the majority of cases. The inclusion of muscle biopsy data improved the accuracy of all subtypes, apart from ADM (94% without muscle biopsy data and 60% with) and JDM (97% without muscle biopsy data and 96% with). Agreement between the EULAR/ACR criteria and the Bohan and Peter criteria was found to be 89% without muscle biopsy data and 93% with biopsy data. External validation in the Euromyositis registry and the Juvenile Dermatomyositis Biomarker Study and Repository revealed sensitivity of 100% for both adult and juvenile populations. A website-based calculator has been developed to allow for further use in other study populations: www.imm.ki.se/biostatistics/calculators/iim.

The criteria have a number of major strengths. Their development methodology included a large IIM population with non-IIM comparators and considered inclusion of a wide variety of candidate variables. The ease of use and provision of a website-based calculator are also major strengths. Drawbacks include the fact that a majority of the development population were white, thus potentially limiting the criteria's validity in Asian and African populations. Further, MRI and electromyography data were only available in 38 and 29% of cases, thus potentially excluding these variables from the criteria only due to missing data. A limitation of subtype identification is the inability to separately identify IMNM and ASS cases. The inclusion of only one myositis-specific autoantibody (anti-Jo-1) in the classification criteria is a major limitation, as recent studies have illustrated the important and distinctive clinical and subtype associations [9–11]. Therefore, the authors have recommended that a future update of the EULAR/ACR criteria should include more IMNM, ADM, hypomyopathic dermatomyositis, juvenile IIM other than JDM and MSA-positive cases, to allow accurate identification.

In summary, the newly developed EULAR/ACR classification criteria for the IIMs provide an accurate method through which clearly defined study populations can be formed, thus potentially improving validity of IIM research.

## DIAGNOSIS

The newly developed classification criteria provide robust methods for identifying IIM cases for research purposes; however, their use is not designed nor recommended for use in clinical practice.

Accurate diagnosis of an IIM is key to appropriate treatment instigation, prognostication and prevention of complications. However, diagnosis and subtype identification in clinical settings can be challenging, in part due to potential multisystem involvement and wide variations between subtype manifestations. Currently, no clear diagnostic criteria for the IIMs exist. However, findings from clinically focused research studies can aid a clinician's diagnostic accuracy, identification of factors associated with prognosis and guide investigation of multisystem involvement.

## CLINICAL FEATURES

Findings from epidemiological and observational studies can inform clinical practice and guide the diagnostic process. A number of observational studies have recently been published and these will be summarized, with a particular focus upon potential clinical applications. Cox *et al.*[12] recently described ‘hiker's feet’, hyperkeratosis of skin of the feet in cases of dermatomyositis. This newly described manifestation was detected in nine dermatomyositis cases out of a large IIM cohort (*N* = 2145). Interestingly, seven of the nine cases also fulfilled criteria for ASS and six cases were positive for anti-Jo-1 antibodies. This study highlights the importance of foot examination in IIM cases when trying to identify cutaneous manifestations. Mamyrova *et al.*[13^▪^] identified that only sun exposure and nonsteroidal anti-inflammatory use were significantly associated with an IIM flare, which highlights the importance of ascertaining a patient's symptom relationship with these factors. Svensson *et al.*[14] recently investigated if there is an association between IIM onset and preceding infections and respiratory tract disease. Preceding infections were significantly more common in IIM cases, than controls: 13 vs. 9%, respectively. They also identified that respiratory tract disease was associated with IIM onset. The authors therefore concluded that infections may increase the risk of IIM development through possible activation of the inflammatory response.

A recent study compared the utility of muscle testing via hand-held dynamometry against manual muscle testing in myositis cases [15]. They reported that hand-held dynamometry was particularly accurate at assessing the strength of single muscle groups, whereas manual muscle testing was only reliable in assessing generalised muscle weakness. Therefore, consideration should be given to the wider use of handheld dynamometry as a method of assessing and quantifying muscle strength and identifying weakness during a diagnostic assessment.

## INTERSTITIAL LUNG DISEASE

Interstitial lung disease (ILD) is an important extramuscular manifestation to consider during the diagnostic process and subsequent assessments. A number of studies have recently investigated risk factors for ILD. A recent study by Schiffenbauer *et al.*[16] has highlighted important relationships between cigarette smoking, ILD risk and clinical and serological manifestations in the IIMs. White IIM cases who had ever smoked were more likely to have polymyositis and test positive for an antisynthetase or anti-Jo-1 autoantibodies; they were also less likely to test positive for antitranscriptional intermediary factor antibody. Whites who had ever smoked were more likely to develop ILD, whereas the risk of ILD was decreased for ever smokers in the African–American population. This complements the finding by Chinoy *et al.*[17] in 2012, who identified an association between smoking and development of anti-Jo-1 antibodies in cases positive for HLA-DRB1^∗^03, thus indicating a genetic interaction with smoking and IIM development. These studies therefore highlight the importance of ascertaining a patient's smoking history due to its potential diagnostic utility and estimation of ILD risk.

Investigation for predictors of poor survival in 497 IIM-associated ILD cases was carried out by Sato *et al.*[18]. They identified that older age of onset (>60 years), raised C-reactive protein, low peripheral capillary oxygen saturation (<95%) and positivity for the antibody against melanoma differentiation-associated gene 5 were all associated with increased mortality risk. Therefore, presence of these factors can aid prognostication. Ang *et al.*[19] reported cutaneous dermatomyositis features associated with ILD development in their cohort of 101 dermatomyositis cases. They reported that the presence of mechanics’ hands was significantly associated with an increased risk of ILD, whereas the presence of Gottron's papules was associated with a significantly reduced risk, thus highlighting the potential prognostic value of pattern of cutaneous manifestations.

## IMAGING

The role of imaging in IIM diagnosis has expanded in recent years and has a number of capabilities. Imaging techniques, such as MRI, can help identify the presence of myositis, delineate its extent and assess treatment response, through serial scans and also help focus appropriate areas for muscle biopsy, limiting the likelihood of a false negative sample.

Studies by both Yao *et al.*[20^▪^] and Andersson *et al.*[21] have recently identified associations between semi-quantitative MRI scores and a number of variables, including physician global assessment, the modified childhood myositis assessment scale and creatine kinase levels in IIM cases; Yao *et al.*[20^▪^] also compared changes in semi-quantitative scores prior to and following rituximab therapy and identified no consistent changes. It could be that the use of such semi-quantitative scoring systems may aid assessment during the diagnostic process; however, further evidence for its particular utility is required.

Pinal-Fernandez *et al.*[22] reported the particular MRI features of IMNM, compared with polymyositis, dermatomyositis, CADM and IBM. They reported that more widespread muscle oedema, atrophy and fat replacement were associated with IMNM and that positivity for the antibody against signal recognition particle was associated with more severe disease, than those that were positive for the antibody against HMG-CoA reductase. Further MRI features in IBM were characterized by Guimaraes *et al.*[23] in 12 biopsy-proven cases. MRI scans of upper and lower limbs were scored for muscle atrophy, fat infiltration and oedema pattern. They concluded that changes were most severe in the lower limbs and the most common abnormality was fat infiltration. Further, they identified that the number of muscles with fat infiltration was statistically associated with disease duration, muscle strength and functional status. Therefore, MRI may be a useful and sensitive method through which IIM subtype can be identified; however, correlation with clinical features, serological status and muscle biopsy examination will complement this.

Focused MRI scanning can identify focal areas on myositis; however, whole-body MRI offers the ability to completely delineate all muscle groups affected and potentially identify secondary organ involvement and the presence of associated malignancy. Whole-body MRI, as opposed to focused imaging, was advocated by Elessawy *et al.*[24^▪^] as the modality of choice due to the ability to detect all muscle groups affected, which may not be evident on clinical examination. Huang *et al.*[25] also reported the utility of whole-body MRI and advocated its use to help identify associated malignancy, extramuscular manifestations, such as ILD and cardiac involvement, and steroid-associated osteonecrosis.

Other imaging modalities also offer utility in IIM diagnosis. Burlina *et al.*[26^▪^] recently aimed to develop and evaluate the capability of ultrasound imaging to automatically identify myositis through ‘machine learning’ and ‘deep learning’ statistical techniques. Machine and deep learning allow the development of computational algorithms that can detect the presence of a disease, thus potentially aiding or superseding human diagnostic skills. The developed algorithm could therefore allow automatic detection of myositis through ultrasound imaging. The developed algorithms displayed accuracies ranging 69–87%. The developed algorithms also enabled distinction between IBM, polymyositis and dermatomyositis. This study therefore identified the capability of automated ultrasound imaging to detect myositis and highlighted potential future innovation.

Computed tomography (CT) scanning offers detailed assessment of the presence of ILD. A recent study by Ungprasert *et al.*[27] investigated the associations between pulmonary function tests and quantitative thoracic high-resolution CT analysis in an IIM population with ILD. After investigating for associations in 110 cases, they concluded that the quantitative measurements correlate well with pulmonary function test values, which included diffusing capacity for carbon monoxide, total lung capacity and oxygen saturations, thus extending patient assessment through CT imaging.

## CARDIAC INVOLVEMENT

The ability to identify cardiac involvement (an uncommon but important extramuscular manifestation) and distinguish from other heart disease has undergone advances recently. The utility of MRI in identifying cardiac involvement has been confirmed in recent years by a small number of studies [28,29]. Recent advances include identification that raised serum levels of troponin I can be used as a reliable indicator of myocardial involvement in the IIMs and can distinguish between active myocardial and skeletal muscle disease [30]. Hughes *et al.*[31] have recently developed a pathway using cardiac troponins to screen for subclinical cardiac disease in IIM patients. Guerra *et al.*[32] aimed to identify the ability of ‘global longitudinal strain’ measurement via echocardiography to identify subclinical systolic impairment in IIM cases. Through investigation of 28 IIM cases and comparison with healthy controls, they concluded that subclinical systolic impairment is common and that the global longitudinal strain method may be useful in identification.

Huber *et al.*[33^▪^] confirmed the ability of MRI to identify inflammatory cardiac disease; however, they identified an inability to differentiate between inflammatory cardiac disease due to IIM or acute viral myocarditis. Differentiation was only possible with MRI examination of the thoracic skeletal muscles.

## CONCLUSION

Accurate case identification is key to IIM research and the recent publication of the EULAR/ACR classification criteria will potentially greatly improve IIM research through accurate case identification and standardization across studies. Clear diagnosis of the IIMs is important to ensure appropriate diagnosis and treatment instigation. Recent advances in knowledge of clinical features will aid the clinician in prognostication, treatment stratification and investigation for multisystem involvement.

## Acknowledgements

None.

### Financial support and sponsorship

This work was supported by Medical Research Council (MRC) UK grant MR/N003322/1. The views expressed in this publication are those of the author(s) and not necessarily those of the NHS, the National Institute for Health Research or the Department of Health.

### Conflicts of interest

There are no conflicts of interest.

## REFERENCES AND RECOMMENDED READING

Papers of particular interest, published within the annual period of review, have been highlighted as:

▪ of special interest
▪▪ of outstanding interest
